# Spatial-temporal parameters during unobstructed walking in people with Parkinson's disease and healthy older people: a public data set

**DOI:** 10.3389/fnagi.2024.1354738

**Published:** 2024-03-28

**Authors:** Tiago Penedo, Carlos Augusto Kalva-Filho, Jônatas Augusto Cursiol, Murilo Henrique Faria, Daniel Boari Coelho, Fabio Augusto Barbieri

**Affiliations:** ^1^Human Movement Research Laboratory (MOVI-LAB), Department of Physical Education, São Paulo State University (Unesp), Bauru, Brazil; ^2^Center for Mathematics, Computation, and Cognition, Federal University of ABC, São Bernardo do Campo, Brazil; ^3^Biomedical Engineering, Federal University of ABC, São Bernardo do Campo, Brazil

**Keywords:** motor control, biomechanics, gait, kinematics, human movement

## Introduction

The literature is consistent: Parkinson's disease (PD) impairs spatial-temporal gait performance. People with PD show shorter step length, larger step width, slower step velocity, and longer step duration (Barbieri et al., 2018; Penedo et al., 2018). In addition, gait of people with PD is characterized by larger spatial-temporal variability and asymmetry (Peterson and Horak, 2016; Simieli et al., 2017; Barbieri et al., 2018). The negative effects of PD on gait are identified in the early disease stages (Rehman et al., 2019; Balaji et al., 2020; Ferreira et al., 2022), worsening with the disease progression (Albani et al., 2014; Pistacchi et al., 2017). The changes in gait parameters are a result of an abnormal increase in GABAergic outputs from the basal ganglia, which causes excessive inhibition in the substantia nigra pars compacta in the midbrain locomotor region (Takakusaki, 2017). Since spatial-temporal gait impairments are directly related to brain degeneration and PD symptoms, spatial-temporal gait changes are an attractive option to help diagnose PD and monitor disease progression. However, most studies comparing spatial-temporal gait parameters between people with PD and neurologically healthy peers include a reduced number of participants.

Public large data sets of spatial-temporal gait parameters in people with PD are relevant to improve the knowledge of gait changes in PD. Beyond increasing the power of the findings in the study, a large public dataset offers the possibility of implementing more robust algorithms to improve the understanding of the effects of PD on gait. However, to our knowledge, there are no published open data sets with spatial-temporal gait parameters of people with PD during unobstructed walking. Previous datasets related to PD's gait present important data about freezing gait characteristics in people with PD (Souza et al., 2022) but not in non-freezing individuals. In addition, given the heterogeneity of the patient's conditions and the importance of highlighting the effects of PD, it is critical to have a matched-neurologically healthy group (e.g., age and sex) to compare and confirm the changes in unobstructed walking.

The purpose of this study, therefore, is to introduce an open data set that contains spatial-temporal gait parameters of people with PD and neurologically healthy olders. The dataset includes (a) demographics such as age, gender, body mass and height, and body mass index, (b) clinical conditions like PD onset, severity and stage, and cognitive assessment; and (c) spatial-temporal gait step and stride parameters such as length, width, duration, velocity, double and single support time, and cadence.

## Methods

The data were acquired in the Human Movement Research Laboratory (MOVI-LAB) at São Paulo State University (Unesp), Bauru, SP, Brazil. The ethics committee from the School of Science at Unesp approved the study protocol (CAAE #78660517.2.0000.5398), and all individuals signed a consent form before the data collection. All measurements in people with PD were performed during ON medication conditions: the individuals had taken dopaminergic medication 1 h before starting the sessions (Araújo-Silva et al., 2022).

### Participants

A sample of 126 individuals (74 females and 52 males) was recruited from 2016 to 2021. It consisted of 63 individuals with PD and 63 age-, body mass-, and body height-matched neurologically healthy individuals. The PD participants were recruited from the Ativa Parkinson's community project at Unesp - Campus Bauru, and the neurologically healthy people from the community. The participants were interviewed to collect their demographic, sociocultural, and overall health conditions. Their ages varied from 52 to 84 years, body masses from 42.0 to 115.3 kg, heights from 1.41 to 1.84 m, and body mass index (BMI) from 16.2 to 46.8 kg/m^2^ (Table 1).

**Table 1:** Demographic, sociocultural, and overall health conditions data of people with PD and neurologically healthy older people (controls).

				**PD**					**Statistics (Controls** × **PD)**	
	**Overall (*n =* 126)**	**Controls (*n =* 63)**	**General (*n =* 63)**	**Mild H&Y**			**Moderate H&Y**			
				**1 (*****n** =* **4)**	**1.5 (*****n** =* **5)**	**2 (*****n** =* **33)**	**2.5 (*****n** =* **14)**	**3 (*****n** =* **7)**	**Value**	***p*** **-value**
M/F (n)	52 M / 74 F	18 M / 45 F	34 M / 29 F	1 M / 3 F	2 M / 3 F	18 M / 15 F	9 M / 5 F	4 M / 3 F	***x*^2^ = 8.383**	**0.004** ^*^
Age (yr)	68.6 ± 6.9	68.3 ± 6.3	68.8 ± 7.5	65.3 ± 10.5	69.4 ± 8.3	68.1 ± 7.1	69.0 ± 7.5	73.1 ± 7.4	t = 0.373	0.710
	[52-84]	[57-81]	[52-84]	[53-74]	[58-79]	[61-84]	[53-79]	[62-84]		
Body mass (kg)	70.7 ± 13.5	72.1 ± 13.2	69.4 ± 13.7	72.7 ± 9.7	79.5 ± 13.5	72.2 ± 11.9	62.7 ± 15.6	60.7 ± 13.3	t = −1.206	0.232
	[42.0-115.3]	[44.9-115.3]	[42.0-98.0]	[63.8-82.2]	[65.1-98.0]	[48.8-95.0]	[42.0-97.0]	[48.0-81.3]		
Height (m)	1.61 ± 0.08	1.60 ± 0.08	1.62 ± 0.09	1.63 ± 0.05	1.64 ± 0.09	1.62 ± 0.09	1.62 ± 0.09	1.58 ± 0.11	t = 1.736	0.088
	[1.41-1.84]	[1.46-1.84]	[1.41-1.76]	[1.60-1.71]	[1.55-1.76]	[1.46-1.76]	[1.46-1.74]	[1.41-1.71]		
BMI (kg/m^2^)	27.3 ± 4.7	28.2 ± 4.7	26.4 ± 4.7	27.3 ± 3.0	29.6 ± 5.7	27.4 ± 4.1	23.7 ± 5.2	24.0 ± 3.4	**t** **=** **−2.187**	**0.033** ^*^
	[16.2-46.8]	[20.0-46.8]	[16.2-37.2]	[24.9-31.3]	[21.8-35.7]	[22.9-37.2]	[16.2-32.8]	[19.0-29.5]		
MMSE (pts)	27.7 ± 2.0	27.9 ± 2.0	27.5 ± 2.0	28.5 ± 1.9	28.0 ± 2.1	27.5 ± 2.1	27.5 ± 1.4	26.4 ± 2.6	t = −1.252	0.215
	[21-30]	[21-30]	[21-30]	[26-30]	[25-30]	[21-30]	[25-30]	[21-28]		
PD onset (yrs)	-	-	5.3 ± 3.7	7.0 ± 5.2	4.4 ± 2.7	5.2 ± 3.6	5.9 ± 4.1	4.9 ± 3.3	-	-
			[1-15]	[2-12]	[1-7]	[2-15]	[2-13]	[2-10]		
H&Y (pts)	-	-	2.1 ± 0.5	-	-	-	-	-	-	-
			[13]							
UPDRS-III (pts)	-	-	27.5 ± 10.6	12.8 ± 2.8	18.6 ± 3.9	27.1 ± 9.9	31.6 ± 8.4	35.4 ± 12.2	-	-
			[10-58]	[10-16]	[13-24]	[19-58]	[14-47]	[19-48]		

Values in bold and the asterisk indicates significant differences between groups.

PD, Parkinson's disease; H&Y, Hoehn and Yahr scale; M, male; F, female; BMI, body mass index; MMSE, Mini-Mental State Examination; UPDRS-III, Unified Parkinson's Disease Rating Scale-part III.

The participants were included if they presented the following criteria:

General:

- Age ≥50 years old;
- Preserved cognitive functions (> 24 points in the Mini-mental State Examination - MMSE, adjusted by the level of education);
- Any vestibular, visual, or somatosensory dysfunctions as self-declared;
- Be able to walk independently.

Only for PD:

- Diagnosis confirmed by a movement disorders specialist, according to the UK Brain Bank criteria;
- Any neurological or physical dysfunctions other than those associated with PD;
- No surgery for PD;
- Be under antiparkinsonian drug treatment for at least 3 months;
- Mild to moderate PD progression (Hoehn and Yahr scale - H&Y from 1 to 3).

### Procedures

The participants were provided with information about the protocol for data collection, and the entire process was conducted in a single session. Following these explanations, the participant signed the informed consent form. The researcher then conducted interviews with the participants to gather information about their demographics, sociocultural backgrounds, and overall health conditions. One movement disorders specialists applied the following scales to verify: (i) Cognitive function screening: MMSE score (Brucki et al., 2003); (ii) PD motor severity: Unified Parkinson's Disease Rating Scale (UPDRS-III) (Goetz et al., 1993); (iii) PD progression: H&Y (Hoehn and Yahr, 1967). After 5 min of restoring in a sitting position, participants walked barefoot three times on an unobstructed path at their own self-selected velocity, wearing comfortable clothes. No freezing of gait episodes happened during the trials.

### Data acquisition and processing

**Figure 1:**
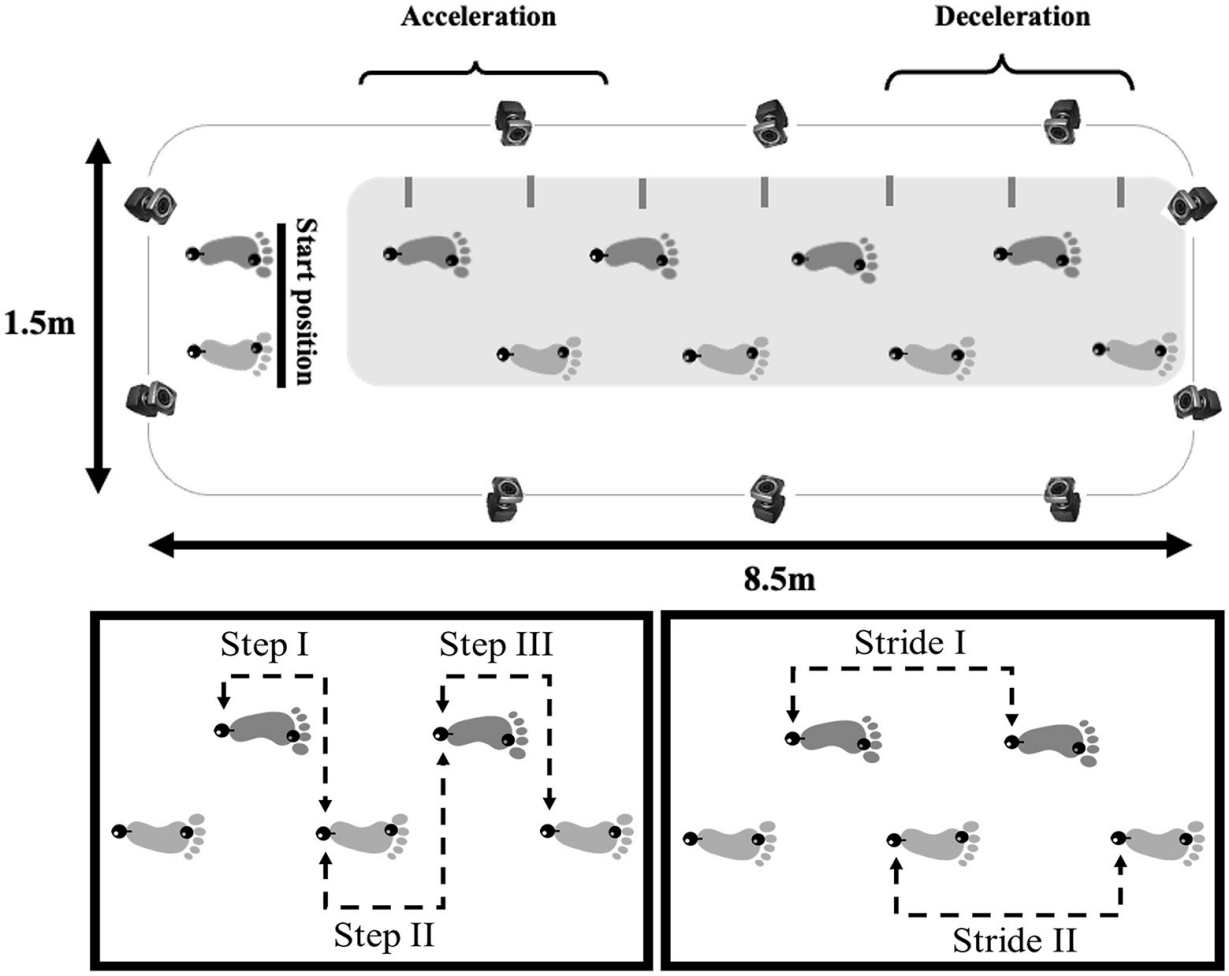
Illustration of the unobstructed path (8.5 × 1.5 m) where participants walked and gait spatial-temporal data of healthy older people and people with PD were collected **(top panel)** with a 3D capture system (represented by cameras) or 2D capture system (represented by a gray rectangular), showing the starting position, acceleration and deceleration phases (steps that were excluded from the analysis). The lower panels show the central three steps **(left panel)** and two strides **(right panel)** analyzed. The black spheres on both feet represent the reflective markers positioned on the calcaneus and second metatarsal.

The gait parameters were acquired using two-dimensional (2D) or three-dimensional (3D) methods:

- 2D: The GAITRite^®^ walkway system (100 Hz) was employed. A carpet (5.74 m long) with pressure sensors was positioned over a 7 m straight segment to capture the gait parameters;
- 3D: Ten Vicon Motion Systems^®^ cameras (100 Hz) were utilized (Figure 1). Two passive reflective markers were placed on each participant's foot (second metatarsal and calcaneus). The acquired data were filtered using a 5th-order low-pass digital Butterworth filter (zero-lag) with a 6 Hz cut-off frequency.

It is important to mention that the use of different kinematic tools did not affect the quality of the data. A previous study (Webster et al., 2005) compared the two systems for measuring both average and individual spatial-temporal gait parameters, revealing an excellent level of agreement between the methods. The intraclass correlation coefficients (ICC) ranged from 0.92 to 0.99.

The gait parameters calculated and their definitions are presented in Supplementary material 1. The parameters were extracted through an algorithm developed in the Matlab software (version 7.10, Mathworks) (Supplementary material 2) and Excel sheets for 3D and 2D data collection, respectively. The participants completed at least seven steps in each trial. The central three steps were analyzed, disregarding the first two steps to minimize gait acceleration and deceleration effects (Figure 1).

## Results

The data is available at the Institutional Repository of Unesp (https://repositorio.unesp.br/items/783cb1b0-d327-45c2-beb3-bf1db1b3f7f1). The dataset contains three types of information: two text files (.csv) with (a) metadata and (b) spatial-temporal processed data for each session, and (c) two text files (.txt) with the instructions and general information contained in .csv files. Descriptive statistics and distribution of each gait parameter contained in the dataset are presented in Supplementary material 3.

### Metadata

The metadata files named Control-Group_gait_data.csv and Parkinson_disease-Group_gait_data.csv contain information from each participant's demographic, sociocultural, and overall health conditions. Here is the coding for the metadata:

For the Control-Group_gait_data.csv file (columns A to G):

A. Participant_code: the code of the participants from #1CG to #63CG.
B. Age: participant's age in years.
C. Gender: gender (F for females or M for males).
D. Body_mass: body mass in kilograms (measured with a calibrated scale).
E. Body_height: height in meters (measured with a calibrated stadiometer).
F. BMI: Body Mass Index, in kilograms per square meter (kg/m^2^).
G. MMSE: Mini-Mental State Examination, in points.

For Parkinson_disease-Group_gait_data.csv file (columns A to J):

A. Participant_code: the code of the participants from #1PD to #63PD.
B. PD_onset: the onset of PD, in years.
C. H&Y: Hoehn and Yahr scale, in points.
D. UPDRS-III: Unified Parkinson's Disease Rating Scale-part III, in points.
E. MMSE: Mini-Mental State Examination, in points.
F. Age: participant's age in years.
G. Gender: gender (F for females or M for males).
H. Body_mass: body mass in kilograms (measured with a calibrated scale).
I. Body_height: height in meters (measured with a calibrated stadiometer).
J. BMI: Body Mass Index, in kilograms per square meter (kg/m^2^).

### Processed data

All spatial-temporal processed data are stored in CSV format and are named by the corresponding participant code (from #1 to #63) and group (PD – Parkinson's disease or C - Controls) plus the number of trials (from 1 to 3). Each file consists of a header of 189 rows (three rows for each participant trial), and 45 columns (27 columns with each variable for three steps and 18 columns with each variable for two strides).

For the Control-Group_gait_data.csv file (columns H to BB) and for Parkinson_disease-Group_gait_data.csv file (columns K to BE):

1. Participant_code: the code of the participants from #1 to #63
2. Trial: trials 1, 2, and 3, for each variable collected.
3. Length _step_1: length for step 1, in centimeters.
4. Length _step_2: length for step 2, in centimeters.
5. Length _step_3: length for step 3, in centimeters.
6. Width _step_1: width for step 1, in centimeters.
7. Width _step_2: width for step 2, in centimeters.
8. Width _step_3: width for step 3, in centimeters.
9. Duration _step_1: duration for step 1, in seconds.
10. Duration _step_2: duration for step 2, in seconds.
11. Duration _step_3: duration for step 3, in seconds.
12. Velocity _step_1: velocity for step 1, in centimeters per second.
13. Velocity _step_2: velocity for step 2, in centimeters per second.
14. Velocity _step_3: velocity for step 3, in centimeters per second.
15. DS_time_step_1: double support time for step 1, in seconds.
16. DS_time_step_2: double support time for step 2, in seconds.
17. DS_time_step_3: double support time for step 3, in seconds.
18. DS _step_1: normalized double support for step 1, in percentage.
19. DS _step_2: normalized double support for step 2, in percentage.
20. DS _step_3: normalized double support for step 3, in percentage.
21. SS_time_step_1: single support time for step 1, in seconds.
22. SS_time_step_2: single support time for step 2, in seconds.
23. SS_time_step_3: single support time for step 3, in seconds.
24. SS _step_1: normalized single support for step 1, in percentage.
25. SS _step_2: normalized single support for step 2, in percentage.
26. SS _step_3: normalized single support for step 3, in percentage.
27. Cadence _step_1: cadence for step 1, in steps per second.
28. Cadence _step_2: cadence for step 2, in steps per second.
29. Cadence _step_3: cadence for step 3, in steps per second.
30. Length _ stride_1: length for stride 1, in centimeters.
31. Length _stride_2: length for stride 2, in centimeters.
32. Width _stride_1: width for stride 1, in centimeters.
33. Width _stride_2: width for stride 2, in centimeters.
34. Duration _stride_1: duration for stride 1, in seconds.
35. Duration _ stride_2: duration for stride 2, in seconds.
36. Velocity_stride _1: velocity for stride 1, in centimeters per second.
37. Velocity_stride _2: velocity for stride 2, in centimeters per second.
38. DS_time_stride _1: double support time for stride 1, in seconds.
39. DS_time_stride _2: double support time for stride 2, in seconds.
40. DS_stride_1: normalized double support for stride 1, in percentage.
41. DS_stride_2: normalized double support for stride 2, in percentage.
42. SS_time_stride_1: single support time for stride 1, in seconds.
43. SS_time_stride_2: single support time for stride 2, in seconds.
44. SS _stride_1: normalized single support for stride 1, in percentage.
45. SS _stride_2: normalized single support for stride 2, in percentage.
46. Cadence_stride_1: cadence for stride 1, in strides per second.
47. Cadence_stride_2: cadence for stride 2, in strides per second.

### Comparison between groups

Demographic, sociocultural, and overall health conditions data were compared by an independent *t*-test (*p* < 0.05 – Table 1). The dependence between gender distribution and group was performed through the Chi-Square (x^2^) test (*p* < 0.05 – Table 1). The groups were different in gender and BMI.

## Discussion

This manuscript provides a publicly available dataset with assessments on the spatial-temporal gait parameters in people with PD and neurologically healthy older people. No other public database provides processed spatial-temporal of unobstructed gait data of people with PD, allowing for straightforward comparisons between steps and strides or between trials. Our database is a valuable resource for PD research. In addition, there is the possibility of performing comparisons by calculating variability (intra-steps or intra-trials, or between participants) and gait asymmetry (steps or strides) between people with PD and neurologically healthy people.

Gait can be implemented to assess health status (Cesari et al., 2005), physical function (Studenski et al., 2003), and quality of life (Ferrucci et al., 2000), while some specific parameters may indicate dementia (Verghese et al., 2007), risk of falls (Maki, 1997), and even risk of early death (Studenski et al., 2011). However, access to data for gait parameters is often scarce. Considering the current research on gait in people with PD and neurologically healthy adults, these measures are relevant to the scientific community. For example, a previous study (Ferreira et al., 2022) used machine learning algorithms based on the spatial-temporal gait parameters variability and asymmetry, demonstrating the potential to distinguish people with PD from matched-healthy people and to identify the PD stages. Hence, through this dataset it is possible to perform several calculations with the spatial-temporal parameters of unobstructed gait (i.e., intra- and inter-trial variability and asymmetry), identifying what happens to gait during the healthy aging process and as a result of the PD.

The spatial-temporal parameter differences during unobstructed gait between people with PD and neurologically healthy people are well-established in the literature. Therefore, matching groups according to their demographic characteristics is important to avoid bias or misinterpreting the results. The demographic, sociocultural, and overall health conditions data from this dataset were compared and only found differences between the groups for gender (*p* < 0.004) and BMI (*p* < 0.033). Since some health and body characteristics (i.e., body composition) are related to gait deficits in people with PD (Barbieri et al., 2022), these factors should be considered when analyzing gait data from people with PD and neurologically healthy people. Regarding the gender differences, it has been pointed out that females and males with PD present specific phenotypes concerning the symptoms of the disease (i.e., postural instability and more severe tremors in females, and more severe rigidity and bradykinesia in males) (Georgiev et al., 2017; Picillo et al., 2017). Therefore, some differences in the gait patterns of people with PD associated with gender are already expected. However, a previous study did not identify gender-related differences in the spatial-temporal gait parameters of people mildly affected by PD (Porta et al., 2019).

Finally, the availability of this public dataset is expected to enable countless investigations and contributions to the unobstructed gait of people with Parkinson's disease (PD) and neurologically healthy individuals. This issue is of great relevance to the scientific community, as it allows for the assessment of physical function, dementia, risk of falls, quality of life, and even the risk of death in these populations. By understanding the neuromotor and biomechanical impairments of gait caused by both the aging process and PD, we may be able to develop new strategies to improve the gait performance of these populations.

## Data availability statement

The datasets presented in this study can be found in online repositories. The names of the repository/repositories and accession number(s) can be found at: https://repositorio.unesp.br/items/783cb1b0-d327-45c2-beb3-bf1db1b3f7f1/Institutional Repository of São Paulo State University.

## Ethics statement

The studies involving humans were approved by Ethics Committee from the School of Science at São Paulo State University. The studies were conducted in accordance with the local legislation and institutional requirements. The participants provided their written informed consent to participate in this study.

## Author contributions

TP: Writing – review & editing, Writing – original draft, Methodology, Data curation, Conceptualization. CK-F: Writing – review & editing. JC: Writing – review & editing, Methodology, Data curation. MF: Writing – review & editing, Methodology, Data curation. DC: Writing – review & editing. FB: Writing – review & editing, Writing – original draft, Supervision, Project administration, Methodology, Funding acquisition, Conceptualization.
