# Supplementary material for: Spatial-temporal parameters during unobstructed walking in people with Parkinson's disease and healthy older people: a public data set
Source: Front Aging Neurosci. 2024 Mar 28;16:1354738. doi: 10.3389/fnagi.2024.1354738 (PMC11007149; doi:10.3389/fnagi.2024.1354738)
Supplement: Supplementary file 1 [file Table_1.docx]

**Supplementary material 1**. Definitions of the spatial-temporal gait parameters are presented. Adapted from Hollman et al. (Hollman et al., 2011).

| Gait parameter | |  | Definition | | |
| --- | --- | --- | --- | --- | --- |
|  |  |  | Step |  | Stride |
| Spatial | Length (cm) |  | Anterior-posterior distance from the calcaneus of one foot to the calcaneus of the opposite foot |  | Anterior-posterior distance between calcaneus of two consecutive footprints of the same foot |
|  | Width (cm) |  | Lateral distance from the calcaneus center of one footprint to the line of progression formed by two consecutive footprints of the opposite foot |  | The mean of the two-step width |
|  |  |  |  |  |  |
| Temporal | Cadence (steps/ or strides/s) |  | Number of steps per second |  | Number of strides per second |
|  | Duration (s) |  | Time elapsed from initial contact of one foot to initial contact of the opposite foot |  | Time elapsed between the initial contacts of two consecutive footfalls of the same foot |
|  | Single Support Time (s) (occurs when only one foot is in contact with the ground) |  | Time elapsed between the last contact of the opposite footfall to the initial contact of the next footfall of the same foot |  | Time elapsed between the last two contacts of the opposite footfall to the initial contacts of the next footfall of the same foot |
|  | Double Support Time (s) (occurs when both feet are in contact with the ground simultaneously) |  | The sum of the duration elapsed during one period of double support in the step |  | The sum of the duration elapsed during two periods of double support in the stride |
|  |  |  |  |  |  |
| Temporophasic | Single Support (%) |  | Single support time normalized to step duration |  | Single support time normalized to stride duration |
|  | Double Support (%) |  | Double support time normalized to step duration |  | Double support time normalized to stride duration |
|  |  |  |  |  |  |
| Spatial-temporal | Velocity (cm/s) |  | Step length divided by the step duration |  | Stride length divided by the stride duration |
