# Supplementary material for: Spatial-temporal parameters during unobstructed walking in people with Parkinson's disease and healthy older people: a public data set
Source: Front Aging Neurosci. 2024 Mar 28;16:1354738. doi: 10.3389/fnagi.2024.1354738 (PMC11007149; doi:10.3389/fnagi.2024.1354738)

**Supplementary material 3.1.** Descriptive statistics of the spatiotemporal parameters observed during the step in healthy controls (C) and people with Parkinson’s disease (PD).

|  |  | Mean (SD) | CI95% | Median (IQR) | Minimal | Q1 | Q3 | Maximal | Skewness | Kurtosis |
| --- | --- | --- | --- | --- | --- | --- | --- | --- | --- | --- |
| Length (cm) | C | 61.82 (6.29) | 60.27 - 63.37 | 62.27 (7.06) | 46.70 | 58.48 | 65.54 | 76.48 | -0.20 | 0.35 |
|  | PD | 55.81 (7.75) | 53.90 - 57.73 | 56.55 (10.42) | 37.54 | 50.68 | 61.10 | 72.32 | -0.17 | -0.49 |
| Width (cm) | C | 13.63 (4.32) | 12.57 - 14.7 | 13.70 (6.27) | 4.39 | 10.35 | 16.62 | 24.49 | 0.40 | -0.09 |
|  | PD | 12.59 (4.23) | 11.55 - 13.63 | 11.90 (4.97) | 3.83 | 10.07 | 15.04 | 26.44 | 0.63 | 0.94 |
| Duration (s) | C | 0.52 (0.05) | 0.5 - 0.53 | 0.51 (0.07) | 0.39 | 0.48 | 0.55 | 0.63 | -0.06 | -0.17 |
|  | PD | 0.53 (0.05) | 0.51 - 0.54 | 0.53 (0.04) | 0.43 | 0.50 | 0.55 | 0.66 | 0.37 | 0.76 |
| Velocity (cm.s^-1^) | C | 121.24 (17.48) | 116.92 - 125.55 | 120.79 (20.93) | 76.61 | 110.88 | 131.81 | 162.29 | 0.09 | 0.22 |
|  | PD | 107.25 (17.95) | 102.82 - 111.68 | 109.11 (21.94) | 66.58 | 96.70 | 118.64 | 159.88 | 0.04 | 0.27 |
| Double support (s) | C | 0.19 (0.05) | 0.18 - 0.20 | 0.19 (0.08) | 0.10 | 0.15 | 0.23 | 0.32 | 0.21 | -0.62 |
|  | PD | 0.20 (0.07) | 0.18 - 0.22 | 0.19 (0.11) | 0.09 | 0.14 | 0.25 | 0.42 | 0.82 | 0.61 |
| Double support (%SC) | C | 12.44 (4.82) | 11.25 - 13.63 | 12.64 (5.73) | 5.36 | 9.37 | 15.10 | 39.38 | 2.75 | 14.67 |
|  | PD | 37.28 (11.3) | 34.49 - 40.07 | 36.92 (20.33) | 17.86 | 27.32 | 47.66 | 66.12 | 0.28 | -0.83 |
| Single support (s) | C | 0.33 (0.06) | 0.31 - 0.34 | 0.33 (0.10) | 0.22 | 0.28 | 0.38 | 0.45 | -0.11 | -1.11 |
|  | PD | 0.33 (0.06) | 0.32 - 0.35 | 0.33 (0.09) | 0.19 | 0.29 | 0.37 | 0.49 | 0.12 | -0.27 |
| Single support (%SC) | C | 63.21 (9.92) | 60.77 - 65.66 | 64.19 (17.22) | 43.78 | 54.44 | 71.66 | 81.17 | -0.01 | -1.19 |
|  | PD | 63.22 (10.69) | 60.58 - 65.86 | 63.59 (19.90) | 43.35 | 52.78 | 72.68 | 82.14 | -0.14 | -1.18 |
| Cadency (Steps.s^-1^) | C | 1.96 (0.2) | 1.91 - 2.01 | 1.95 (0.27) | 1.58 | 1.81 | 2.08 | 2.57 | 0.61 | 0.48 |
|  | PD | 1.92 (0.18) | 1.87 - 1.96 | 1.90 (0.17) | 1.51 | 1.83 | 2.00 | 2.36 | 0.33 | 0.60 |
| Standard deviation |  |  |  |  |  |  |  |  |  |  |
| Length (cm) | C | 2.83 (1.73) | 2.40 - 3.26 | 2.48 (1.41) | 0.74 | 1.73 | 3.15 | 8.83 | 1.81 | 3.39 |
|  | PD | 2.70 (1.18) | 2.41 – 3.00 | 2.62 (1.57) | 0.75 | 1.81 | 3.38 | 6.37 | 0.96 | 1.17 |
| Width (cm) | C | 3.07 (1.49) | 2.70 - 3.43 | 2.77 (1.75) | 0.78 | 1.94 | 3.69 | 7.99 | 1.14 | 1.83 |
|  | PD | 2.49 (1.17) | 2.20 - 2.78 | 2.18 (1.63) | 0.97 | 1.59 | 3.21 | 5.94 | 0.93 | 0.20 |
| Duration (s) | C | 0.05 (0.24) | -0.01 - 0.11 | 0.02 (0.01) | 0.01 | 0.01 | 0.03 | 1.93 | 7.91 | 62.74 |
|  | PD | 0.02 (0.01) | 0.02 - 0.03 | 0.02 (0.01) | 0.01 | 0.01 | 0.03 | 0.08 | 1.88 | 4.96 |
| Velocity (cm.s^-1^) | C | 6.59 (3.18) | 5.80 - 7.37 | 6.26 (3.96) | 1.27 | 4.11 | 8.07 | 15.51 | 0.77 | 0.20 |
|  | PD | 6.34 (2.54) | 5.71 - 6.96 | 6.38 (2.78) | 0.94 | 4.72 | 7.50 | 15.61 | 1.02 | 2.53 |
| Double support (s) | C | 0.02 (0.01) | 0.02 - 0.02 | 0.02 (0.01) | 0.00 | 0.01 | 0.02 | 0.05 | 0.92 | 0.26 |
|  | PD | 0.02 (0.01) | 0.02 - 0.02 | 0.02 (0.01) | 0.01 | 0.01 | 0.03 | 0.06 | 1.42 | 2.22 |
| Double support (%SC) | C | 17.62 (5.21) | 16.33 - 18.90 | 17.65 (8.51) | 4.77 | 13.65 | 22.16 | 27.68 | -0.18 | -0.82 |
|  | PD | 3.81 (2.18) | 3.27 - 4.35 | 3.31 (2.15) | 1.29 | 2.40 | 4.55 | 12.96 | 2.11 | 5.60 |
| Single support (s) | C | 0.03 (0.01) | 0.02 - 0.03 | 0.02 (0.02) | 0.01 | 0.02 | 0.03 | 0.06 | 1.12 | 0.98 |
|  | PD | 0.03 (0.01) | 0.02 - 0.03 | 0.02 (0.01) | 0.01 | 0.02 | 0.03 | 0.08 | 1.45 | 2.53 |
| Single support (%SC) | C | 3.80 (2.01) | 3.30 - 4.29 | 3.46 (2.28) | 0.49 | 2.39 | 4.67 | 11.16 | 1.27 | 2.10 |
|  | PD | 3.87 (2.48) | 3.26 - 4.49 | 3.31 (2.15) | 1.29 | 2.40 | 4.55 | 16.88 | 2.95 | 12.10 |
| Cadency (Steps.s^-1^) | C | 0.08 (0.05) | 0.07 - 0.09 | 0.07 (0.06) | 0.02 | 0.04 | 0.10 | 0.31 | 2.28 | 9.12 |
|  | PD | 0.08 (0.05) | 0.07 - 0.10 | 0.07 (0.04) | 0.03 | 0.05 | 0.10 | 0.30 | 2.22 | 5.53 |
| Variability |  |  |  |  |  |  |  |  |  |  |
| Length (cm) | C | 4.61 (2.72) | 3.94 - 5.28 | 3.95 (2.43) | 1.03 | 2.77 | 5.20 | 12.05 | 1.42 | 1.50 |
|  | PD | 5.00 (2.55) | 4.37 - 5.63 | 4.43 (2.61) | 1.32 | 3.24 | 5.85 | 13.65 | 1.40 | 2.37 |
| Width (cm) | C | 23.54 (11.68) | 20.65 - 26.42 | 21.1 (14.11) | 7.55 | 15.54 | 29.65 | 71.86 | 1.68 | 4.60 |
|  | PD | 20.72 (8.38) | 18.65 - 22.79 | 20.08 (13.64) | 7.79 | 14.47 | 28.10 | 37.90 | 0.23 | -1.08 |
| Duration (s) | C | 9.85 (47.03) | -1.76 - 21.47 | 3.47 (2.70) | 1.07 | 2.36 | 5.05 | 376.84 | 7.91 | 62.72 |
|  | PD | 4.34 (2.60) | 3.70 - 4.98 | 3.55 (2.36) | 1.43 | 2.83 | 5.19 | 15.23 | 2.16 | 5.98 |
| Velocity (cm.s^-1^) | C | 5.49 (2.66) | 4.83 - 6.14 | 5.17 (3.51) | 0.98 | 3.47 | 6.98 | 13.05 | 0.87 | 0.61 |
|  | PD | 6.09 (2.72) | 5.41 - 6.76 | 5.83 (2.98) | 0.88 | 4.28 | 7.26 | 15.76 | 1.16 | 2.04 |
| Double support (s) | C | 11.21 (7.55) | 9.35 - 13.07 | 8.34 (9.63) | 2.13 | 5.74 | 15.36 | 34.36 | 1.29 | 1.16 |
|  | PD | 11.33 (6.82) | 9.65 - 13.01 | 8.76 (10.57) | 2.42 | 6.44 | 17.01 | 31.59 | 0.90 | -0.14 |
| Double support (%SC) | C | 146.5 (17.32) | 142.22 – 150.78 | 147.9 (0.81) | 12.11 | 147.71 | 148.52 | 157.84 | -7.77 | 61.23 |
|  | PD | 11.2 (6.81) | 9.52 - 12.88 | 8.86 (9.12) | 2.55 | 6.20 | 15.32 | 32.66 | 1.27 | 1.32 |
| Single support (s) | C | 8.15 (4.26) | 7.10 - 9.21 | 7.73 (4.29) | 2.47 | 5.22 | 9.51 | 24.33 | 1.30 | 2.57 |
|  | PD | 8.24 (4.08) | 7.23 - 9.25 | 7.23 (4.01) | 2.86 | 5.58 | 9.59 | 26.61 | 2.11 | 6.64 |
| Single support (%SC) | C | 6.1 (3.27) | 5.29 - 6.90 | 5.52 (3.87) | 0.60 | 3.80 | 7.68 | 20.22 | 1.63 | 4.69 |
|  | PD | 6.28 (4.27) | 5.22 - 7.33 | 5.16 (3.21) | 1.84 | 3.81 | 7.02 | 29.46 | 3.34 | 15.09 |
| Cadency (Steps.s^-1^) | C | 3.93 (2.18) | 3.39 - 4.47 | 3.46 (2.57) | 1.08 | 2.35 | 4.93 | 14.06 | 1.94 | 6.36 |
|  | PD | 4.33 (2.55) | 3.70 - 4.96 | 3.46 (2.36) | 1.42 | 2.84 | 5.20 | 14.86 | 1.98 | 4.95 |

SD: Standard deviation; CI95%: confidence interval of 95%; IQR: Interquartile range; Q1: First quartile; Q3: third quartile; SC: step cycle.

**Supplementary material 3.2.** Descriptive statistics of the spatiotemporal parameters observed during the stride in healthy controls (C) and people with Parkinson’s disease (PD).

|  |  | Mean (SD) | CI95% | Median (IQR) | Minimal | Q1 | Q3 | Maximal | Skewness | Kurtosis |
| --- | --- | --- | --- | --- | --- | --- | --- | --- | --- | --- |
| Length (cm) | C | 123.51 (12.73) | 120.37 - 126.66 | 125.39 (13.90) | 93.73 | 116.80 | 130.70 | 153.47 | -0.15 | 0.30 |
|  | PD | 111.73 (15.73) | 107.85 - 115.62 | 113.24 (22.80) | 74.58 | 100.08 | 122.88 | 144.85 | -0.21 | -0.52 |
| Width (cm) | C | 13.67 (4.43) | 12.58 - 14.77 | 13.05 (6.31) | 4.96 | 10.30 | 16.61 | 24.98 | 0.46 | -0.18 |
|  | PD | 12.60 (4.30) | 11.56 - 13.68 | 12.10 (4.80) | 3.84 | 10.02 | 14.85 | 25.87 | 0.58 | 0.63 |
| Duration (s) | C | 1.03 (0.10) | 1.01 - 1.05 | 1.02 (0.15) | 0.78 | 0.96 | 1.10 | 1.27 | -0.05 | -0.17 |
|  | PD | 1.10 (0.10) | 1.03 - 1.08 | 1.10 (0.10) | 0.85 | 1.00 | 1.10 | 1.33 | 0.39 | 0.85 |
| Velocity (cm.s^-1^) | C | 121.21 (17.63) | 116.85 - 125.56 | 120.85 (20.57) | 77.04 | 110.61 | 131.19 | 167.43 | 0.16 | 0.33 |
|  | PD | 107.10 (18.10) | 102.67 - 111.6 | 108.50 (24.00) | 66.46 | 94.94 | 118.97 | 159.49 | 0.00 | 0.17 |
| Double support (s) | C | 0.38 (0.11) | 0.35 - 0.41 | 0.38 (0.15) | 0.20 | 0.30 | 0.45 | 0.63 | 0.18 | -0.60 |
|  | PD | 0.40 (0.10) | 0.36 - 0.42 | 0.40 (0.20) | 0.18 | 0.28 | 0.48 | 0.75 | 0.56 | -0.19 |
| Double support (%GC) | C | 36.89 (9.95) | 34.43 - 39.34 | 35.70 (18.34) | 18.96 | 28.17 | 46.51 | 55.82 | 0.01 | -1.21 |
|  | PD | 36.60 (10.60) | 33.98 - 39.23 | 36.50 (19.60) | 17.56 | 27.14 | 46.71 | 56.76 | 0.19 | -1.13 |
| Single support (s) | C | 0.65 (0.12) | 0.62 - 0.68 | 0.67 (0.21) | 0.42 | 0.55 | 0.75 | 0.89 | -0.16 | -1.08 |
|  | PD | 0.70 (0.10) | 0.64 - 0.69 | 0.70 (0.20) | 0.38 | 0.58 | 0.74 | 0.96 | 0.06 | -0.14 |
| Single support (%GC) | C | 63.11 (9.95) | 60.66 - 65.57 | 64.30 (18.34) | 44.18 | 53.49 | 71.83 | 81.04 | -0.01 | -1.21 |
|  | PD | 63.40 (10.60) | 60.77 - 66.02 | 63.50 (19.60) | 43.24 | 53.29 | 72.86 | 82.44 | -0.19 | -1.13 |
| Cadency (Steps.s^-1^) | C | 0.98 (0.10) | 0.96 - 1.01 | 0.98 (0.14) | 0.79 | 0.91 | 1.05 | 1.28 | 0.59 | 0.43 |
|  | PD | 1.00 (0.10) | 0.94 - 0.98 | 1.00 (0.10) | 0.76 | 0.91 | 1.00 | 1.17 | 0.31 | 0.65 |
| Standard deviation |  |  |  |  |  |  |  |  |  |  |
| Length (cm) | C | 3.59 (3.09) | 2.82 - 4.35 | 2.99 (2.28) | 0.85 | 1.78 | 4.06 | 18.18 | 3.15 | 11.97 |
|  | PD | 3.43 (2.03) | 2.93 - 3.94 | 3.08 (2.11) | 0.99 | 2.02 | 4.14 | 11.75 | 1.81 | 4.75 |
| Width (cm) | C | 2.09 (1.24) | 1.79 - 2.40 | 1.82 (1.04) | 0.40 | 1.28 | 2.32 | 6.73 | 1.72 | 3.84 |
|  | PD | 1.90 (1.20) | 1.57 - 2.16 | 1.40 (1.70) | 0.38 | 1.00 | 2.71 | 6.26 | 1.29 | 1.75 |
| Duration (s) | C | 0.03 (0.02) | 0.02 - 0.03 | 0.02 (0.02) | 0.01 | 0.01 | 0.04 | 0.13 | 2.23 | 7.69 |
|  | PD | 0.00 (0.00) | 0.02 - 0.03 | 0.00 (0.00) | 0.00 | 0.02 | 0.03 | 0.08 | 1.33 | 1.22 |
| Velocity (cm.s^-1^) | C | 4.84 (3.12) | 4.07 - 5.61 | 4.06 (4.27) | 0.64 | 2.55 | 6.82 | 14.38 | 0.92 | 0.35 |
|  | PD | 4.40 (2.30) | 3.84 – 5.00 | 3.80 (2.50) | 1.47 | 2.86 | 5.38 | 13.15 | 1.48 | 2.81 |
| Double support (s) | C | 0.03 (0.01) | 0.02 - 0.03 | 0.02 (0.02) | 0.01 | 0.02 | 0.03 | 0.07 | 1.28 | 1.96 |
|  | PD | 0.00 (0.00) | 0.02 - 0.04 | 0.00 (0.00) | 0.00 | 0.02 | 0.04 | 0.13 | 2.16 | 6.63 |
| Double support (%GC) | C | 2.41 (1.40) | 2.06 - 2.75 | 2.14 (1.44) | 0.21 | 1.45 | 2.89 | 6.69 | 1.22 | 1.45 |
|  | PD | 2.60 (1.90) | 2.17 - 3.11 | 2.10 (1.80) | 0.28 | 1.53 | 3.30 | 11.67 | 2.39 | 7.96 |
| Single support (s) | C | 0.03 (0.02) | 0.03 - 0.04 | 0.03 (0.02) | 0.01 | 0.02 | 0.04 | 0.11 | 1.76 | 3.88 |
|  | PD | 0.00 (0.00) | 0.03 - 0.04 | 0.00 (0.00) | 0.00 | 0.02 | 0.04 | 0.12 | 1.79 | 5.33 |
| Single support (%GC) | C | 2.41 (1.40) | 2.06 - 2.75 | 2.14 (1.44) | 0.21 | 1.45 | 2.89 | 6.69 | 1.22 | 1.45 |
|  | PD | 2.60 (1.90) | 2.17 - 3.11 | 2.10 (1.80) | 0.28 | 1.53 | 3.30 | 11.67 | 2.39 | 7.96 |
| Cadency (Steps.s^-1^) | C | 0.03 (0.02) | 0.02 - 0.03 | 0.02 (0.02) | 0.00 | 0.01 | 0.03 | 0.16 | 3.59 | 19.15 |
|  | PD | 0.00 (0.00) | 0.02 - 0.03 | 0.00 (0.00) | 0.00 | 0.01 | 0.03 | 0.08 | 1.80 | 3.22 |
| Variability |  |  |  |  |  |  |  |  |  |  |
| Length (cm) | C | 2.88 (2.22) | 2.33 - 3.43 | 2.47 (2.06) | 0.59 | 1.48 | 3.53 | 11.90 | 2.47 | 7.56 |
|  | PD | 3.17 (2.06) | 2.66 - 3.68 | 2.66 (1.94) | 0.74 | 1.86 | 3.80 | 11.79 | 2.08 | 6.01 |
| Width (cm) | C | 16.09 (8.71) | 13.94 - 18.24 | 14.19 (9.61) | 2.27 | 9.90 | 19.51 | 45.17 | 1.15 | 1.34 |
|  | PD | 15.20 (8.20) | 13.15 - 17.22 | 13.90 (12.20) | 2.96 | 8.22 | 20.37 | 34.38 | 0.64 | -0.41 |
| Duration (s) | C | 2.71 (2.10) | 2.19 - 3.23 | 1.93 (2.10) | 0.47 | 1.40 | 3.50 | 13.88 | 2.75 | 11.93 |
|  | PD | 2.50 (1.60) | 2.14 - 2.91 | 1.90 (1.40) | 0.34 | 1.48 | 2.90 | 7.48 | 1.48 | 1.84 |
| Velocity (cm.s^-1^) | C | 4.01 (2.56) | 3.38 - 4.64 | 3.29 (3.79) | 0.49 | 2.06 | 5.85 | 12.15 | 0.95 | 0.53 |
|  | PD | 4.20 (2.30) | 3.66 - 4.81 | 3.70 (2.50) | 1.27 | 2.78 | 5.31 | 13.50 | 1.56 | 3.32 |
| Double support (s) | C | 7.46 (4.95) | 6.24 - 8.68 | 5.99 (5.74) | 1.44 | 3.89 | 9.62 | 25.29 | 1.61 | 2.75 |
|  | PD | 8.60 (6.70) | 6.89 - 10.22 | 6.40 (7.10) | 0.85 | 4.26 | 11.39 | 37.56 | 2.14 | 6.16 |
| Double support (%GC) | C | 7.00 (4.85) | 5.80 - 8.19 | 5.42 (4.78) | 1.10 | 3.69 | 8.47 | 25.74 | 1.69 | 3.27 |
|  | PD | 7.90 (6.40) | 6.31 - 9.45 | 5.40 (6.80) | 1.01 | 3.72 | 10.51 | 34.52 | 2.21 | 6.09 |
| Single support (s) | C | 5.11 (3.32) | 4.28 - 5.93 | 4.31 (3.49) | 0.86 | 2.89 | 6.38 | 19.58 | 1.86 | 5.12 |
|  | PD | 5.00 (2.90) | 4.26 - 5.68 | 4.70 (2.60) | 0.69 | 3.39 | 5.95 | 17.28 | 1.71 | 5.02 |
| Single support (%GC) | C | 3.93 (2.39) | 3.34 - 4.53 | 3.40 (2.53) | 0.26 | 2.23 | 4.75 | 13.12 | 1.33 | 2.45 |
|  | PD | 4.20 (2.90) | 3.50 - 4.95 | 3.50 (2.60) | 0.38 | 2.41 | 4.97 | 17.64 | 2.17 | 6.87 |
| Cadency (Steps.s^-1^) | C | 2.71 (2.16) | 2.17 - 3.24 | 1.90 (2.05) | 0.47 | 1.40 | 3.45 | 14.61 | 3.01 | 14.06 |
|  | PD | 2.50 (1.60) | 2.14 - 2.93 | 1.90 (1.40) | 0.34 | 1.47 | 2.89 | 8.48 | 1.63 | 2.73 |

SD: Standard deviation; CI95%: confidence interval of 95%; IQR: Interquartile range; Q1: First quartile; Q3: third quartile; GC: gait cycle.

**Supplementary material 3.3.** Violin plots showing the distribution of dataset spatial-temporal gait step parameters in people with Parkinson’s disease (PD – blue violins) and healthy controls (C – red violins).


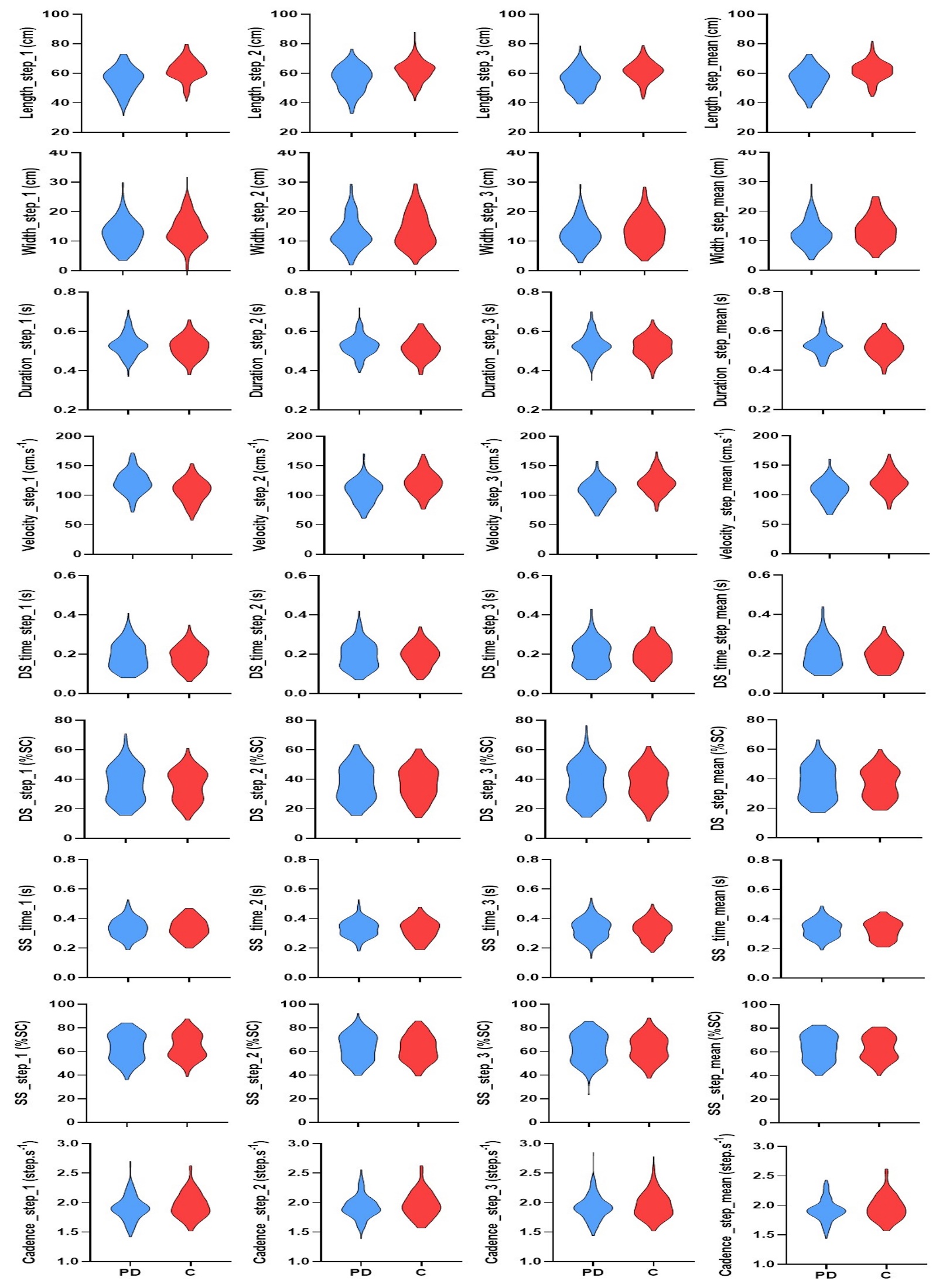


**Supplementary material 3.4.** Violin plots showing the distribution of dataset spatial-temporal gait stride parameters in people with Parkinson’s disease (PD – blue violins) and healthy controls (C – red violins).


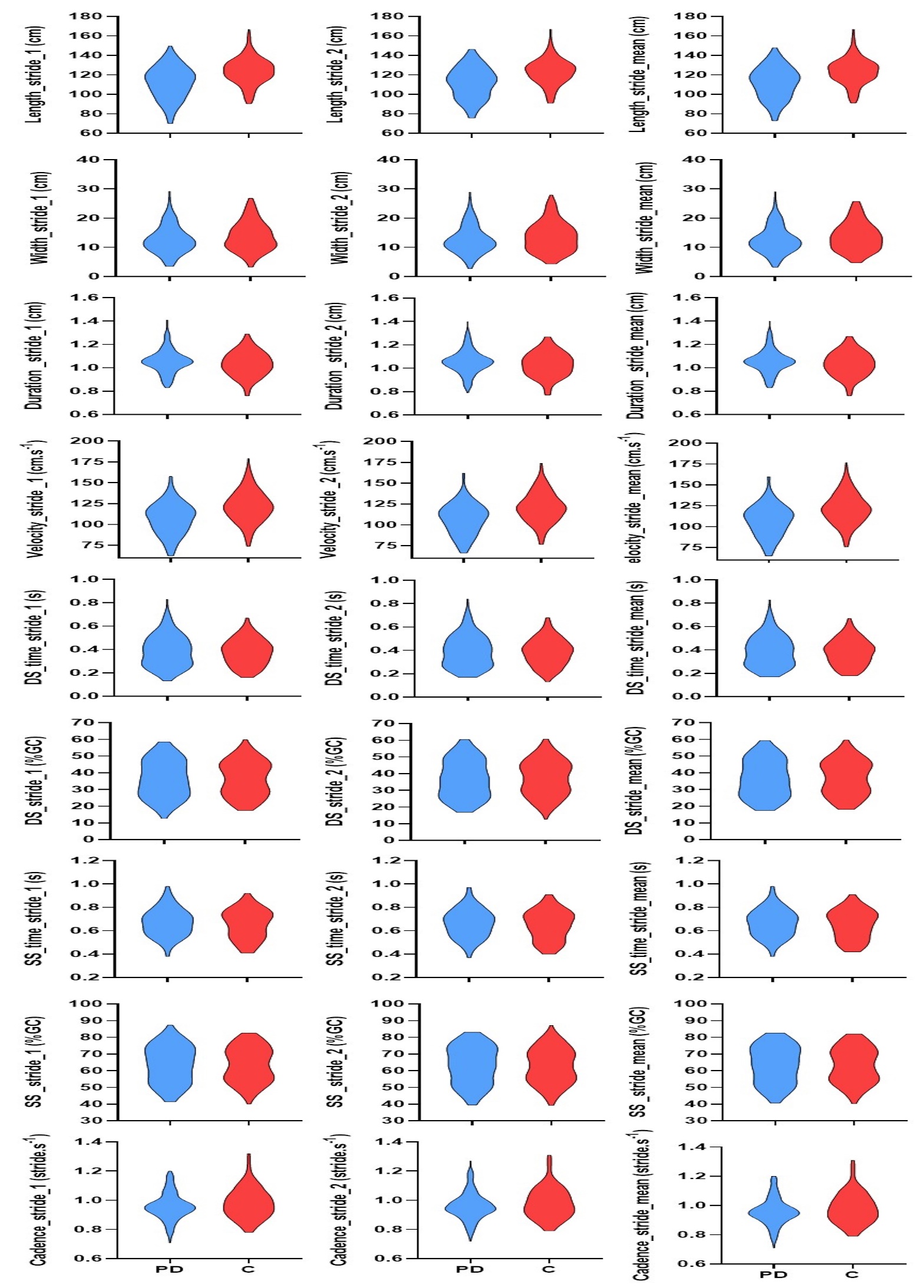

Supplement: Supplementary file 2 [file Data_Sheet_1.docx]
