# Supplementary material for: Spatial-temporal parameters during unobstructed walking in people with Parkinson's disease and healthy older people: a public data set
Source: Front Aging Neurosci. 2024 Mar 28;16:1354738. doi: 10.3389/fnagi.2024.1354738 (PMC11007149; doi:10.3389/fnagi.2024.1354738)
Supplement: Supplementary file 3 [file Data_Sheet_2.docx]

% The script was created by Prof. Fabio A. Barbieri.

% The script finds the heel contact and toe-off automatically and calculates the spatial-temporal parameters during overground walking. The data calculation considers 3 steps and 2 strides.

% The data can't have gap. Axe x - represents medial-lateral movement, Axe y - represents anterior-posterior movement, Axe z - represents vertical movement.

% It is possible to run the script consecutively.

% For this script, it is necessary to have landmarkers on right and left calcaneus and second metatartus

%%%%%%%%%%%%%%% Starting the script %%%%%%%%%%%%%%%%%%

continue = 'y';

while continue == 'y';

clear all

% defining data sample

freq = 100; %data sample frequency

S = warning('OFF');

%%%%%%%%%%%%% Loading the trial %%%%%%%%%%%%%%%%%%%

trial = input ('Fill with the name of the trial (e.g., trial_01_walk): ','s');

data = [trial, '.txt'];

eval ([' load ' data]);

trial_final=[trial];

eval (['time = ' trial_final ' (:,1);']); % frames

eval (['trial_walk = ' trial_final ' (:,2:13);']); % 2:13 represents the columns of each landmark: 2:4 - left calcaneus, 5:7 - left metatarsus; 8:10 - right calcaneus; 11:13 - right metatarusus;

%%%%%%%%%%% Defining walking cycle (5 steps) %%%%%%%%%%%%%%%

figure(1);

plot(trial_walk(:,2), 'r')

hold on;

plot(trial_walk(:,5), 'r')

plot(trial_walk(:,8), 'b')

plot(trial_walk(:,11), 'b')

legend('left calcaneus', 'left metatarsus', 'right calcaneus', 'right metatarusus','Location', 'Best')

hold off;

title ('Please mark starting and ending of the trial considering 3 steps. Please start the cycle considering the black line');

xlabel('Time (frames)');

ylabel('horizontal displacement');

[xv,yv]=ginput(2);

time1 = time(xv(1):xv(2),:);

trial_walk_final = trial_walk(xv(1):xv(2),:);

%%%%%%%%%%%%%%%%%% Interpolating the data for frequency 200Hz according the recommedation of doi: 10.1016/j.gaitpost.2006.05.016 %%%%%%%%%%%%%%%%%%%%%%

nlin = size(trial_walk_finall,1);

frame = [1:nlin1]';

xx = [1:0.5:nlin];

ncol = size(trial_walk_final,2);

for i = 1:ncol;

trial_walk_final1(i,:)=spline(frame,trial_walk_final(:,i),xx);

i = i+1;

end

trial_walk_final1 = trial_walk_final';

nlin2 = size(trial_walk_final,1);

%%%%%%%%%%%%%% Naming the landmarkers %%%%%%%%

cal_left = trial_walk_final1(:,1:3);

met_left = trial_walk_final1(:,4:6);

cal_right = trial_walk_final1(:,7:9);

met_right = trial_walk_final1(:,10:12);

%%%% Calculating the linear velocity and acceleration for each landmarker to define the steps %%%%%%

for i = 2:nlin2;

vel_cal_righty(:,i) = (cal_right(i,2)-cal_right(i-1,2))/(1/200);

vel_cal_rightx(:,i) = (cal_right(i,1)-cal_right(i-1,1))/(1/200);

vel_cal_rightz(:,i) = (cal_right(i,3)-cal_right(i-1,3))/(1/200);

vel_met_righty(:,i) = (met_right(i,2)-met_right(i-1,2))/(1/200);

vel_met_rightx(:,i) = (met_right(i,1)-met_right(i-1,1))/(1/200);

vel_met_rightz(:,i) = (met_right(i,3)-met_right(i-1,3))/(1/200);

vel_cal_lefty(:,i) = (cal_left(i,2)-cal_left(i-1,2))/(1/200);

vel_cal_leftx(:,i) = (cal_left(i,1)-cal_left(i-1,1))/(1/200);

vel_cal_leftz(:,i) = (cal_left(i,3)-cal_left(i-1,3))/(1/200);

vel_met_lefty(:,i) = (met_leftq(i,2)-met_leftq(i-1,2))/(1/200);

vel_met_leftx(:,i) = (met_leftq(i,1)-met_leftq(i-1,1))/(1/200);

vel_met_leftz(:,i) = (met_left(i,3)-met_left(i-1,3))/(1/200);

i = i+1;

end

vel_cal_righty = velcal_righty';

vel_cal_rightx = velcal_rightx';

vel_cal_rightz = velmet_rightz';

vel_met_righty = vel_met_righty';

vel_met_rightx = vel_met_rightx';

vel_met_rightz = velmet_rightz';

vel_cal_lefty = velcal_lefty';

vel_cal_leftx = velcal_leftx';

vel_cal_leftz = velcal_leftz';

vel_met_lefty = velmet_lefty';

vel_met_leftx = velmet_leftx';

vel_met_leftz = velmet_leftz';

nlin3 = size(velcdiry,1);

for i = 2:nlin3;

acel_cal_righty(:,i) = (vel_cal_righty(i,1)-vel_cal_righty(i-1,1))/(1/200);

acel_cal_rightx(:,i) = (vel_cal_rightx(i,1)-vel_cal_rightx(i-1,1))/(1/200);

acel_cal_rightz(:,i) = (vel_cal_rightz(i,1)-vel_cal_rightz(i-1,1))/(1/200);

acel_met_righty(:,i) = (vel_met_righty(i,1)-vel_met_righty(i-1,1))/(1/200);

acel_met_rightx(:,i) = (vel_met_rightx(i,1)-vel_met_rightx(i-1,1))/(1/200);

acel_met_rightz(:,i) = (vel_met_rightz(i,1)-vel_met_rightz(i-1,1))/(1/200);

acel_cal_leftx(:,i) = (vel_cal_leftx(i,1)-vel_cal_leftx(i-1,1))/(1/200);

acel_cal_lefty(:,i) = (vel_cal_lefty(i,1)-vel_cal_lefty(i-1,1))/(1/200);

acel_cal_leftz(:,i) = (vel_cal_leftz(i,1)-vel_cal_leftz(i-1,1))/(1/200);

acel_met_lefty(:,i) = (vel_met_lefty(i,1)-vel_met_lefty(i-1,1))/(1/200);

acel_met_leftx(:,i) = (vel_met_leftx(i,1)-vel_met_leftx(i-1,1))/(1/200);

acel_met_leftz(:,i) = (vel_met_leftz(i,1)-vel_met_leftz(i-1,1))/(1/200);

i = i+1;

end

nlin4=size(celcdiry,2);

acel_cal_righty = [acel_cal_righty(1,3:nlin4)]';

acel_cal_rightx = [acel_cal_rightx(1,3:nlin4)]';

acel_cal_rightz = [acel_cal_rightz(1,3:nlin4)]';

acel_met_righty = [acel_met_righty(1,3:nlin4)]';

acel_met_rightx = [acel_met_rightx(1,3:nlin4)]';

acel_met_rightz = [acel_met_rightz(1,3:nlin4)]';

acel_cal_lefty = [acel_cal_lefty(1,3:nlin4)]';

acel_cal_leftx = [acel_cal_leftx(1,3:nlin4)]';

acel_cal_leftz = [acel_cal_leftz(1,3:nlin4)]';

acel_met_lefty = [acel_met_leftty(1,3:nlin4)]';

acel_met_leftx = [acel_met_lefttx(1,3:nlin4)]';

acel_met_leftz = [acel_met_leftz(1,3:nlin4)]';

%%%%% Finding heel contact and toe-off %%%%

figure(2);

[pkshr,locshr] = findpeaks(-acel_cal_righty,'MinPeakDistance',150);

time = [1:size(cal_right)]';

plot(cal_right(:,2),'b.');

hold on;

plot(acel_cal_righty, 'k')

title ('Mark 3 first heel right contacts (minimum value)');

plot(time(locscr),cal_right(locscr,2),'ok');

xlabel('Time (frames)');

ylabel('calcaneus right');

hold off;

%%%Checking automatic marking%%%

check = input ('Typing 1 if correct, or any other number for correcting manually:','s');

if check=='1';

xcr(1,1)=locscr(1);

xcr(2,1)=locscr(2);

xcr(3,1)=locscr(3);

ycr(1,1)=cal_right(locscr(1),2);

ycr(2,1)=cal_right(locscr(2),2);

ycr(3,1)=cal_right(locscr(3),2);

else

[xcr,ycr]=ginput(3); %when necessary to mark mannually

end

figure(3);

[pkshl,locshl] = findpeaks(-acel_cal_lefty,'MinPeakDistance',150);

time = [1:size(acel_cal_left)]';

plot(acel_cal_left(:,2),'r.');

hold on;

plot(acel_cal_lefty,'k');

title ('Mark 3 first heel right contacts (minimum value)');

plot(time(locscl),cal_left(locscl,2),'ok');

xlabel('Time (frames)');

ylabel('calcaneus left');

hold off;

%%%Checking automatic marking%%%

check = input ('Typing 1 if correct, or any other number for correcting manually:','s');

if check=='1'

xcl(1,1)=locscl(1);

xcl(2,1)=locscl(2);

xcl(3,1)=locscl(3);

ycl(1,1)=cal_leftq(locscl(1),2);

ycl(2,1)=cal_leftq(locscl(2),2);

ycl(3,1)=cal_left(locscl(3),2);

else

[xcl,ycl]=ginput(3); %when necessary to mark mannually

end

figure(4);

[pksmetd,locsmetd] = findpeaks(acel_met_righty,'MinPeakDistance',150);

time = [1:size(met_right)]';

plot(met_right(:,2),'b.');

hold on;

plot(acel_met_righty, 'k')

title ('Mark 3 first toe-off (maximum value)');

plot(time(locsmr),met_right(locsmr,2),'ok');

ylabel('metatarsus right');

hold off;

%%%Checking automatic marking%%%

check = input ('Typing 1 if correct, or any other number for correcting manually:','s');

if check=='1';

xmr(1,1)=locsmr(1);

xmr(2,1)=locsmr(2);

xmr(3,1)=locsmr(3);

ymr(1,1)=met_right(locsmr(1),2);

ymr(2,1)=met_right(locsmr(2),2);

ymr(3,1)=met_right(locsmr(3),2);

else

[xmr,ymr]=ginput(3);

end

figure(5);

[pksml,locsml] = findpeaks(acel_met_lefty,'MinPeakDistance',150);

time = [1:size(met_left)]';

plot(met_left(:,2),'r.');

hold on;

plot(acel_met_left,'k');

title ('Mark 3 first toe-off (maximum value)');

plot(time(locsml),met_left(locsml,2),'ok');

xlabel('Time (frames)');

ylabel('metatarsus left');

hold off;

%%%Checking automatic marking%%%

check = input ('Typing 1 if correct, or any other number for correcting manually:','s');

if check=='1';

xml(1,1)=locsml(1);

xml(2,1)=locsml(2);

xml(3,1)=locsml(3);

yml(1,1)=met_left(locsmete(1),2);

yml(2,1)=met_left(locsmete(2),2);

yml(3,1)=met_left(locsmete(3),2);

else

[xml,yml]=ginput(3);

end

%%%%%%%%% Grouping the data for heel contact and toe-off %%%%%%%%%%

xcal_right = [round(xcr(1,1)); round(xcr(2,1)); round(xcr(3,1))];

xmet_right =[round(xmr(1,1)); round(xmr(2,1));round(xmr(3,1))];

xcal_left = [round(xcl(1,1)); round(xcl(2,1));round(xcl(3,1))];

xmet_left = [round(xml(1,1)); round(xml(2,1));round(xml(3,1))];

% finding the values in the data

calright1 = cal_right(abs(xcal_right(1)),1:3);

calright2 = cal_right(abs(xcal_right(2)),1:3);

calright3 = cal_right(abs(xcal_right(3)),1:3);

metright1 = met_right(abs(xmet_right(1)),1:3);

metright2 = met_right(abs(xmet_right(2)),1:3);

metright3 = met_right(abs(xmet_right(3)),1:3);

calleft1 = cal_left(abs(xcal_left(1)),1:3);

calleft2 = cal_left(abs(xcal_left(2)),1:3);

calleft3 = cal_left(abs(xcal_left(3)),1:3);

metleft1 = met_left(abs(xmet_left(1)),1:3);

metleft2 = met_left(abs(xmet_left(2)),1:3);

metleft3 = met_left(abs(xmet_left(3)),1:3);

%%%%%%%%%%%%%% Calculating the outcomes %%%%%%%%%%%%%%%

% step lenght (cm)

sl3 = abs(metleft1(1,2)-metright2(1,2))*100;

sl2 = abs(metright2(1,2)-metleft2(1,2))*100;

sl1 = abs(metleft2(1,2)-metright3(1,2))*100;

sl = [sl3 sl2 sl1];

% stride lenght (cm)

stl2 = abs(metleft1(1,2)-metleft2(1,2))*100;

stl1 = abs(metright2(1,2)-metright3(1,2))*100;

stl = [sl2 sl1];

% step width (cm)

sw3 = abs(metleft1(1,1)-metright2(1,1))*100;

sw2 = abs(metright2(1,1)-metleft2(1,1))*100;

sw1 = abs(metleft2(1,1)-metright3(1,1))*100;

sw = [sw3 sw2 sw1];

% stride width (cm)

stw2 = ((abs(metleft1(1,1)-metleft2(1,1))*100)+(abs(metright2(1,1)-metleft2(1,1))*100))/2;

stw1 = ((abs(metright2(1,1)-metleft2(1,1))*100)+(abs(metleft2(1,1)-metright3(1,1))*100))/2;

stw = [stw2 stw1];

% step duration (s)

freqi = 200; %interpolated sample frequency

sd3 = (abs(xmetright(2,1) - xmetleft(1,1)))/freqi;

sd2 = (abs(xmetleft(2,1) - xmetright(2,1)))/freqi;

sd1 = (abs(xmetright(3,1) - xmetleft(2,1)))/freqi;

sd = [sd3 sd2 sd1];

% stride duration (s)

std2 = (abs(xmetright(2,1) - xmetright(3,1)))/freqi;

std1 = (abs(xmetleft(1,1) - xmetleft(2,1)))/freqi;

std = [std2 std1];

% step velocity (cm/s)

sv3 = sl3/sd3;

sv2 = sl2/sd2;

sv1 = sl1/sd1;

sv = [sv3 sv2 sv1];

% stride velocity (cm/s)

stv2 = stl2/std2;

stv1 = stl1/std1;

stv = [stv2 stv1];

% double support period - step (s)

ds3 = abs(xcalright(1,1)-xmetleft(1,1))/freqi;

ds2 = abs(xcalleft(2,1)-xmetright(2,1))/freqi;

ds1 = abs(xcalright(2,1)-xmetleft(2,1))/freqi;

ds = [ds3 ds2 ds1];

% double support period - stride (s)

dst2 = (abs(xcalright(1,1)-xmetleft(1,1))/freqi)+(abs(xcalleft(2,1)-xmetright(2,1))/freqi);

dst1 = (abs(xcalleft(2,1)-xmetright(2,1))/freqi)+(abs(xcalright(2,1)-xmetleft(2,1))/freqi);

dst = [dst2 dst1];

steps = [sl sw sd sv ds];
strides = [stl stw std stv dst];

% clearing some variables

clear a*; clear nl*; clear frame*; clear xx*; clear tenfil*; clear o*;

clear ce*; clear ve*; clear tu*; clear x*;

%%%%%%%%%%%%%%%% Saving the outcomes %%%%%%%%%%%%%%%

arq_ascii=[tentativ, 'steps' , 'strides' , '.txt'];

eval(['save -ascii ', arq_ascii , ' steps']);

eval(['save -ascii ', arq_ascii , ' strides']);

close all

% continuing to run the trials - y = continue, n - no continue

continue=input('Do you want to run a next trial? (y or n):','s');

end
